# A model for selective visual attention predicts information routing

**DOI:** 10.1186/1471-2202-14-S1-P310

**Published:** 2013-07-08

**Authors:** Daniel Harnack, Udo A Ernst, Klaus R Pawelzik

**Affiliations:** 1 Institut für Theoretische Physik, Universität Bremen, Bremen, Germany

## 

A remarkable feature of the brain is its ability to flexibly process information in a context-dependent manner. For example, selective attention allows to focus on relevant information, and to ignore distracting features of a visual scene. Here we develop a model to investigate a putative neural mechanism by which attention might dynamically change the functional configuration of networks in visual cortex.

Since efferents from multiple neurons in an early visual area converge onto the same neuron in a higher area, synaptic input induced by an attended object should be more efficient in driving the post-synaptic neuron than an input induced by a simultaneously presented, but unattended object. In fact, it has been found that the firing rate of a post-synaptic neuron in such a situation is similar to its activity when the unattended stimulus is absent. However, if no attention is directed while two stimuli are present, the firing rate of the post-synaptic neuron is approximately at the mean of the rates at which it would fire if each of two stimuli were presented alone (biased competition [1,2]). Attention is thus assumed to selectively promote information transfer between visual areas. Recent experiments support this idea by showing that V4 local field potentials are correlated with a temporal modulation of the attended stimulus and not with the temporal modulation of a non-attended stimulus in the same receptive field (gating [3]).

Several models were proposed to explain the effect of biased competition and information routing [4,5]. However, here we construct a biophysically plausible model that specifically reproduces both experimental results within the same framework. It comprises four populations of recurrently coupled networks of conductance-based spiking neurons with lateral inhibition. Upon activation, these populations engage in oscillatory activity. The first two networks correspond to a lower visual area with non-overlapping receptive fields, which both project to the third and the fourth network corresponding to higher area populations with larger receptive fields. We assume attention to manifest as an additional input to one lower area population. Deployment of attention consequently results in a bias of firing rates between the populations in the lower area. This bias induces a phase relation between the lower and the higher area that is more suitable for information transmission from the attended stimulus. We show that this mechanism is capable to explain both, the biased competition effect of the firing rate, and selective gating of information flow. We thus propose a combination of cortical network topology, neuronal population dynamics, and selective additional input as putative neural underpinnings of attention.

## References

[B1] Moran J Desimone R Selective attention gates visual processing in the extrastriate cortex Science 1985 229 782 784 10.1126/science.4023713 4023713

[B2] Reynolds JH Chelazzi L Desimone R Competitive mechanisms subserve attention in macaque areas V2 and V4 J Neurosci 1999 19 1736 1753 10024360 10.1523/JNEUROSCI.19-05-01736.1999 PMC6782185

[B3] Grothe I Neitzel SD Rotermund D Mandon S Linke M Ernst UA Pawelzik KR Kreiter AK Interareal gamma-band synchronization in primate ventral visual pathway underlies signal routing during selective attention Program No 913.18.2012 Neuroscience Meeting Planner 2012 New Orleans, LA: Society for Neuroscience Online

[B4] Zeitler M Fries P Gielen S Biased competition through variations in amplitude of gamma-oscillations J Comput Neurosci 2008 25 89 107 10.1007/s10827-007-0066-2 18293071 PMC2441488

[B5] Montijn JS Klink PC van Wezel RJA Divisive normalization and neuronal oscillations in a single hierarchical framework of selective visual attention Front Neural Circuits 2012 6 22 22586372 10.3389/fncir.2012.00022 PMC3343306

